# Jinyuan 601 a Novel High-Protein Soybean Variety with Improved Agronomic Traits and Nutritional Quality

**DOI:** 10.3390/life15091414

**Published:** 2025-09-08

**Authors:** Xinyu Wei, Xiaoguang Yu, Xiangjin Chen, Shaobin Cui, Jieyin Cui, Ran Wei, Henan Diao, Honglei Ren, Wencheng Lu, Xiaodong Tang

**Affiliations:** 1 Heihe Branch Institute, Heilongjiang Academy of Agricultural Sciences, Heihe 164399, China; weixinyu2001@163.com (X.W.); 1366456190@163.com (X.Y.); sset755@163.com (S.C.); cuijieyin@163.com (J.C.); 17545529703@163.com (R.W.);; 2 Soybean Research Institute, Heilongjiang Academy of Agriculture Sciences, Harbin 150086, China; renhonglei2022@163.com

**Keywords:** *Glycine max*, cultivar development, protein content, agronomic traits, crop improvement, food security

## Abstract

The global demand for high-protein soybeans is rapidly increasing, driven by the growing popularity of healthy foods and plant-based protein products. To address this demand, a novel high-protein soybean variety, Jinyuan 601, was developed through a systematic breeding program. This study details the breeding process, agronomic characteristics, and performance evaluation of Jinyuan 601, which was derived from a cross between Heihe YX10-534 (female parent) and Heihe No. 45 (male parent). The variety was selected over multiple generations (F_2_–F_7_) and stabilized as Heihe 18-250, demonstrating excellent quality, upright stalks, and resistance to diseases and pests. Jinyuan 601 exhibits a protein content of 43.66% and a fat content of 17.21%, meeting the standard for high-protein soybeans (≥43% protein). It has a growth period of 111 days, with a plant height of 93.2 cm, and shows moderate resistance to soybean mosaic virus (SMV). Yield trials conducted over two years (2021–2022) produced an average of 2292 kg ha^−1^, representing a 3.0% increase over the control variety (Huajiang No. 2). This variety holds significant potential for applications in health foods, plant-based products, and sustainable agriculture, contributing to food security and reducing reliance on soybean imports.

## 1. Introduction

The soybean [*Glycine max* (L.) Merr.] is a commercially crucial leguminous crop that has been cultivated for over 6000 years, originating in East Asia and now representing one of the world’s most important agricultural commodities [1,2]. Global soybean production has expanded dramatically, with cultivation spanning six continents and involving more than 170 nations in international trade, generating an anticipated market value of USD 155 billion by 2024 [3,4,5]. The crop serves as a cornerstone of modern agriculture, providing approximately 69% of global plant protein meal and 30% of vegetable oil consumption for both human nutrition and livestock feed [4,6].

Soybean seeds are nutritionally dense, containing substantial quantities of protein (35–45%), oil (17–22%), carbohydrates (30–35%), dietary fiber, vitamins (B, C, and E), and essential minerals, including potassium, phosphorus, and iron [4,7]. This exceptional nutritional profile has positioned soybeans as a crucial component in addressing global food security challenges, particularly as the world population is projected to reach 10 billion by 2050 and dietary preferences increasingly shift toward plant-based protein sources [5,8]. The versatility of soybean protein, with its excellent gelling and emulsifying properties, has made it indispensable in food processing industries worldwide, supporting the production of meat alternatives, dairy substitutes, and functional food ingredients [9,10,11].

The global demand for high-protein soybeans continues to accelerate, driven by expanding health food markets, plant-based product development, and growing consumer awareness of sustainable protein sources [12,13]. Commercial high-protein soybeans, defined as varieties containing ≥43% protein on a dry weight basis, command premium prices and serve specialized market segments, including human food applications, high-quality animal feeds, and protein concentrate manufacturing [14,15]. These varieties provide superior amino acid profiles compared to conventional soybeans, offering complete protein sources that rival animal proteins in nutritional quality while supporting environmentally sustainable production systems [14,16,17].

Soybean seeds contain a significant concentration of lipids and proteins, serving as both a nutrient reservoir for seedling development and a primary raw material for the extraction of soy protein and oil in the food processing sector [18,19,20]. As a result, the enhancement of soybean quality, particularly in terms of its protein and oil content, has attracted considerable attention. Comprehensive studies have demonstrated that both genetic and environmental factors influence the quality attributes of soybeans [21,22,23,24]. In China’s three primary ecological regions—the North, Huang-Huai-Hai, and the South—soybean cultivars exhibit a north-to-south gradient, characterized by an increase in protein content and a corresponding decrease in oil content. High-crude-protein-content soybeans are predominantly found in the Yangtze River basin and the southwestern hilly regions, whereas soybeans with elevated crude oil content are primarily located in the northeastern and northwestern portions of China [25,26]. The observed variances in fat formation are purportedly linked to regional disparities in environmental parameters, including temperature, precipitation, diurnal temperature fluctuations, and sunshine exposure. The oil content in a single soybean variety may fluctuate by more than 1% due to variations in weather conditions and cultivation practices across different areas [27,28]. Utilizing data from 2005 to 2018, Wang et al. [29] conducted a quality analysis of Neidou 4, the primary soybean variety grown in Inner Mongolia’s leading production region, and determined that temperature and precipitation were significant meteorological factors affecting protein content, with temperature being the sole principal factor influencing oil content. The Northeast, characterized by ample sunlight, notable diurnal temperature fluctuations, moderate precipitation, and growth-period temperatures, is an ideal environment for cultivating high-oil soybean types [30,31]. Nevertheless, high-protein cultivars flourish in the northern and central regions of China. The impact of latitude and topography on soybean quality arises from the cumulative influences of light, temperature, water, and nutrients [32,33].

In major soybean-producing regions worldwide, including the United States, Brazil, Argentina, and China, environmental variation significantly impacts both yield and quality traits [34,35]. Regional studies have demonstrated that temperature and precipitation patterns during critical growth phases, particularly seed filling, profoundly influence final protein accumulation and oil content [34,35]. The northeastern regions of China, characterized by shorter growing seasons, distinct diurnal temperature fluctuations, and moderate precipitation, present unique opportunities for high-protein soybean development due to environmental conditions that favor protein synthesis over oil accumulation [34,35].

This study aimed to develop and comprehensively evaluate a novel high-protein soybean variety (Jinyuan 601) that combines superior protein content (≥43%) with competitive agronomic performance, environmental stability, and disease resistance, thereby providing a valuable cultivar for protein-focused production systems and contributing to global food security.

## 2. Materials and Methods

### 2.1. Plant Material and Breeding Program

The high-protein soybean cultivar Jinyuan 601 was developed through a targeted breeding program at the Heihe Branch of the Heilongjiang Academy of Agricultural Sciences. Plant materials used in this study were obtained from the breeding program at the Heihe Branch of the Heilongjiang Academy of Agricultural Sciences (Heilongjiang Province, China). No specific permissions were required for the use of these materials, as they were provided through formal research collaboration with the breeding program. Formal identification of the plant specimens was performed at the Heilongjiang Academy of Agricultural Sciences. All experimental research on the plants complied with the institutional guidelines of the Heihe Branch of the Heilongjiang Academy of Agricultural Sciences.

### 2.2. Breeding Methodology and Cultivar Development

**Figure 1:**
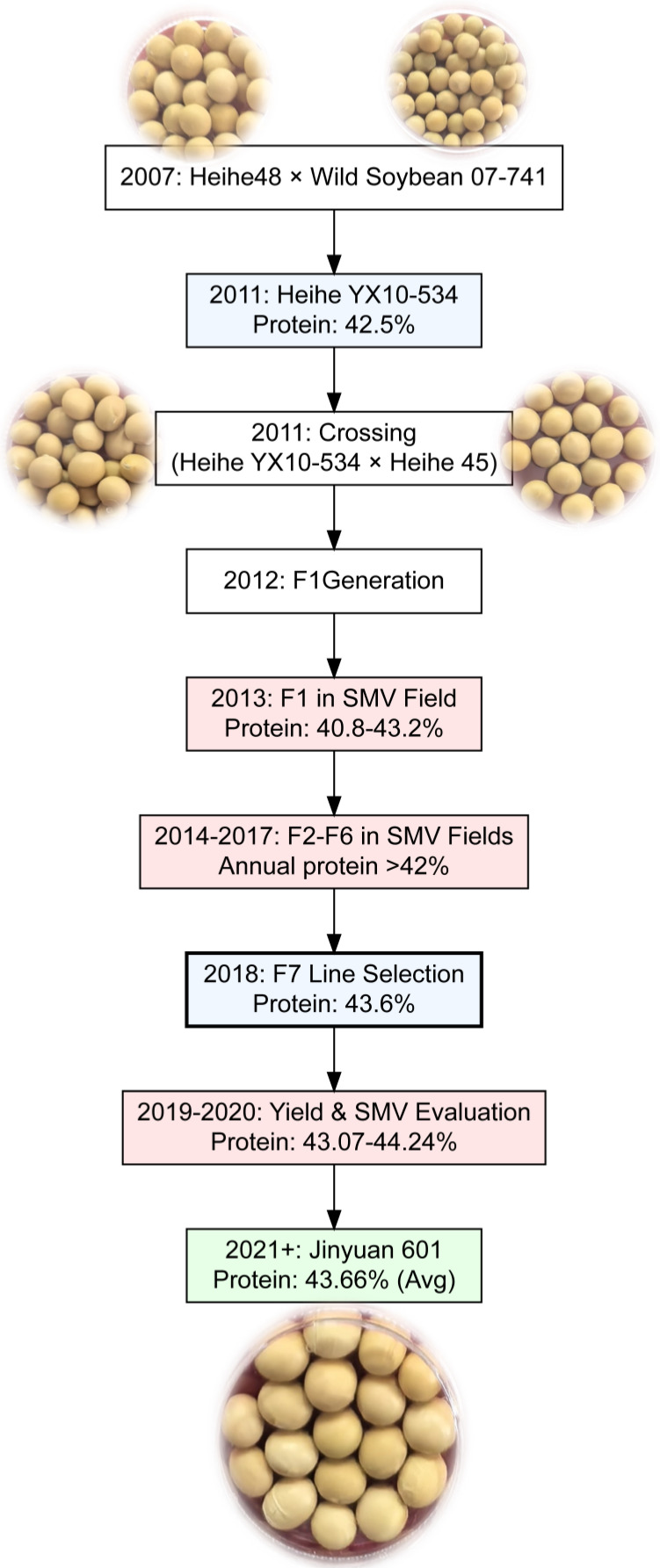
Breeding of the soybean cultivar Jinyuan 601 using a pedigree breeding method.

The breeding process began in 2007 with the creation of an intermediate parent, Heihe YX10-534, derived from a cross between the cultivated variety Heihe 48 and the wild soybean accession 07-741 (Figure 1). This parental line was selected for its high protein content (42.5%) and resistance to Soybean Mosaic Virus (SMV), as confirmed through systematic screening. In 2011, Heihe YX10-534 was crossed with Heihe 45 (a progeny of *Beifeng No. 11* × *Heihe No. 26*) to combine high-protein traits with agronomic stability. The F_1_ generation (2012) was grown under controlled conditions, and pseudo-hybrids were eliminated. From 2013 to 2017, the population was advanced from F_2_ to F_6_ generations using the single-seed descent method under SMV-infested field conditions (Figure 1). Rigorous selection was applied for protein content (consistently greater than 42%) and SMV resistance, with protein levels quantified annually using near-infrared spectroscopy (NIRS). By 2018, the F_7_ generation had yielded the stable line Heihe 18-250, which exhibited a protein content of 43.6%. From 2019 to 2020, the line underwent yield trials and repeated SMV resistance evaluations, demonstrating protein levels of 43.07–44.24% and stable agronomic performance. The cultivar, later designated as Jinyuan 601, maintained an average protein content of 43.66% in subsequent multi-environment trials conducted from 2021 onwards (Figure 1).

### 2.3. Experimental Sites and Environmental Conditions

Field trials were carried out during the 2021 and 2022 growing seasons at nine locations in Northeast China. All trials took place in different areas within Northeast China. The regional trials were held at nine sites—Sun Wu, Longmen, Lingnan, Jiagedaqi, Jianbian, Dougouzi, Huma, Arongqi, and Guli—in 2021 and 2022, with Huajiang No. 2 serving as the control. Three replicates were established for each location, with approximately 10–20 m^2^ allocated per plot in the regional field trials. The production trials were conducted at nine locations in 2021 and 2022, using Qinong 5 as the control, with ~200 m^2^ per plot. Huajiang No. 2 is a local high-yielding cultivar that is moderately resistant to SMV. The experimental sites represent the primary soybean-producing regions of Northeast China and exhibit distinct agroecological characteristics. The soils are predominantly black (Chernozem), loamy, and sandy loam, typical of Heilongjiang Province. During the growing season (late May to mid-September), mean daily temperatures ranged from 16.9 to 19.2 °C, with seasonal ranges between 6 and 31 °C, and precipitation totals of 400–470 mm, depending on location and year (Table 1).

### 2.4. Seed Composition Analysis

#### 2.4.1. Protein Content Determination

Protein content was determined using near-infrared spectroscopy (NIRS DS 2500, FOSS, Hilleroed, Denmark), calibrated against the Kjeldahl method (AOAC Official Method 988.05) [36]. Seed samples were ground to a uniform particle size (<1 mm) using a laboratory mill (Cyclotec 1093, FOSS, Hilleroed, Denmark), and NIRS spectra were collected in duplicate for each sample. For Kjeldahl reference values, samples (1.0 g) were digested in concentrated sulfuric acid (15 mL) with copper catalyst tablets (Kjeltabs CT, FOSS, Hilleroed, Denmark) at 420 °C for 2 h, followed by steam distillation and titration using a Kjeltec 8400 analyzer (FOSS, Hilleroed, Denmark). The NIRS calibration model exhibited a high correlation coefficient (R^2^ > 0.95) with the reference method, ensuring accurate protein quantification. Protein content was calculated using a nitrogen-to-protein conversion factor of 6.25.

#### 2.4.2. Determination of Soybean Seed Oil Content

The oil content of dried soybean seeds was quantitatively evaluated using NIRS DS 2500 (FOSS, Hilleroed, Denmark), calibrated against Soxhlet extraction [36]. This non-destructive spectroscopic method enables rapid and precise assessment of seed composition parameters by analyzing specific wavelength absorption patterns in the near-infrared region (1100–2500 nm). The NIRS instrument was calibrated using reference samples analyzed by the Soxhlet extraction method (AOAC Method 920.85). Each sample underwent triplicate measurements to ensure analytical precision and reliability, with the arithmetic mean used as the definitive value for statistical analysis.

### 2.5. Soybean Mosaic Virus Resistance Evaluation

Resistance to the Soybean Mosaic Virus (SMV) was evaluated through artificial inoculation using strain SC3, a prevalent pathogenic strain in Northeast China. At the V2 growth stage, plants were mechanically inoculated with SMV-SC3 by rubbing carborundum-dusted leaves with infected sap, following protocols described by Bachkar et al. [37]. Disease severity was scored 21 days post-inoculation using a 0–5 scale: 0 = no symptoms, 1 = mild mosaic, 2 = moderate mosaic, 3 = severe mosaic, 4 = mosaic with leaf distortion, 5 = severe mosaic and leaf distortion with stunting [37]. Lines with scores ≤ 2 were classified as resistant. Three independent replicates were conducted to confirm resistance stability. While controlled greenhouse studies would provide more standardized conditions, field-based SMV screening reflects natural infection pressure and environmental variability that cultivars encounter in commercial production systems.

### 2.6. Agronomic Evaluation

Trait assessments were conducted after plants reached full maturity, using 10 randomly selected soybean plants from each plot for comprehensive evaluation. These plants were carefully harvested and transported to the laboratory for detailed analysis of key yield-related traits. The main stem node number (MSNN) was determined by meticulously counting all nodes along the main stem of each sampled plant and then calculating the average across all 10 plants to ensure statistical robustness. Plant height (PH) was measured precisely from the soil surface to the uppermost growing point of the main stem using a standardized measuring tape. Measurements were taken for all 10 plants and subsequently averaged. Pod number per plant (PPP) was assessed by thoroughly counting all pods, including both fully mature and developing immature pods, on each of the 10 sampled plants, with the total value divided by 10 to obtain a representative average. Seeds per plant (SPP) were assessed by thoroughly counting all seeds on each of the 10 sampled plants. The total number of seeds was then used to calculate the seed weight per plant (SWP), with the total value divided by 10 to obtain a representative average. First pod height (PODH) was estimated from the soil to the first pod on the plant. Days to maturity were recorded as the number of days from seedling emergence to physiological maturity (R8 stage), with Heihe 18-250 averaging 108 days. Plant architecture was characterized by an upright growth habit (Type II growth type), with stem lodging resistance scored on a 1–5 scale (1 = completely upright; 5 = completely lodged). Seed quality traits included hilum color (light yellow), seed shape (spherical), and 100-seed weight (HSW) measured from three independent harvests.

### 2.7. Statistical Analysis

Prior to ANOVA analysis, all datasets were tested for normality using the Shapiro–Wilk test (α = 0.05). Non-normally distributed data underwent appropriate transformations (log, square root, or Box–Cox transformation) to meet ANOVA assumptions. Homogeneity of variance was confirmed using Levene’s test.

Statistical analyses were performed using SPSS Statistics 26.0 (IBM Corp., Armonk, NY, USA). Analysis of variance (ANOVA) was conducted using a mixed-effects model with location and year as random effects and genotype as a fixed effect. Treatment means were separated using Fisher’s Least Significant Difference (LSD) test at *p* < 0.05 significance level.

The statistical model used was as follows:

Yijk = μ + Gi + Lj + Yk + GL(ij) + GY(ik) + LY(jk) + GL Y(ijk) + εijk

where

Yijk = observed response

μ = overall mean

Gi = genotype effect (i = 1, 2)

Lj = location effect (j = 1, 2, …, 9)

Yk = year effect (k = 1, 2)

GL(ij) = genotype × location interaction

GY(ik) = genotype × year interaction

LY(jk) = location × year interaction

GLY(ijk) = genotype × location × year interaction

εijk = residual error

Coefficient of variation (CV%) was calculated to assess experimental precision, with CV values < 15% considered acceptable for field trials.

## 3. Results

### 3.1. Analysis of Variance for Soybean Yield Across Environments and Seasons

The analysis of variance revealed highly significant environmental effects on soybean yield (Table 2), with ecological factors accounting for the majority of variation in the dataset. The environmental term (ENV) demonstrated substantial influence on yield differences across locations (F = 815, *p* < 0.001 ***), indicating strong genotype-by-environment interactions. Environmental effects accounted for 94% of the total variation (Sum Sq = 1.81 × 10^4^), emphasizing the dominant role of location-specific factors in determining yield performance. Replication within environments showed no significant effect (F = 1.22, *p* = 0.184 NS), confirming adequate experimental precision and uniform field conditions within locations. However, the interaction between seasons and environments was highly significant (F = 30.1, *p* < 0.001 ***), demonstrating that environmental effects varied considerably between growing years. The model partitioned environmental effects by season, revealing that both 2021 (F = 554, *p* < 0.001 ***) and 2022 (F = 472, *p* < 0.001 ***) contributed significantly to yield variation, with slightly stronger effects observed in 2021. The residual variance remained relatively low (Mean Sq = 2.28), indicating good model fit and acceptable experimental precision. The coefficient of variation (CV = 5.87%) falls within acceptable limits for field trials, while the ratio of maximum to minimum mean square (MSR+/MSR− = 2.69) indicates moderate heterogeneity of variance across treatments. The overall mean yield across environments and seasons was 25.7 units, with environmental effects demonstrating nearly 1000-fold-greater influence than seasonal effects alone (F = 0.0393, *p* = 0.848 NS).

### 3.2. Variability in Agronomic and Quality Traits of Soybean Cultivars

The analysis of key agronomic and quality traits revealed distinct patterns of variability across soybean cultivars (Table 3). HSW showed moderate variation (CV = 9.81%) with values ranging from 14 to 21 g/100 seeds (mean = 16.8 g). Plant architecture traits displayed varying levels of variability, with MSNN showing intermediate variation (CV = 14.4%, range = 9–18 nodes) and PH remaining relatively consistent (CV = 11.6%, mean = 93.4 cm). Reproductive traits exhibited the greatest variability, especially SPP, which showed extreme variation (CV = 85.4%, range = 9–125 seeds), followed by PPP (CV = 40.2%) and SWP (CV = 43.4%). PODH also exhibited substantial variation (CV= 24.8%, range= 8.2–25.3 cm), indicating different branching patterns among cultivars.

Seed quality traits exhibited remarkably low variability, with oil content (mean = 16.8%) and protein content (mean = 43.9%) displaying minimal variation (CV = 3.24% and 1.54%, respectively). The tight ranges for these quality parameters (oil: 15.9–18%; protein: 42.1–44.8%) indicate firm genetic control of these traits compared to the more environmentally sensitive yield components. Standard errors for all traits remained relatively small compared to their means, reflecting adequate sampling precision in the study. The confidence intervals suggest that while yield components may respond strongly to environmental factors, seed composition traits remain stable across growing conditions. These patterns highlight the potential for simultaneously improving both stable quality traits and plastic yield components in soybean breeding programs.

### 3.3. Phenological Development and Yield Performance of Jinyuan 601 Soybean Cultivar Across Different Geographical Locations

The phenological development and yield performance of Jinyuan 601 across nine geographical locations demonstrated significant environmental responsiveness (Table 4). Growing season duration varied considerably among locations, ranging from 97 days at Longmen to 118 days at Huma, with an average of 108.7 days. Relative to control varieties, Jinyuan 601 exhibited variable maturity timing, requiring 1–7 additional days at most locations, though Arongqi showed 2 days earlier maturity. Yield performance revealed substantial location-dependent variation, spanning from 1402.5 kg ha^−1^ at Jiagedaqi to 2592.0 kg ha^−1^ at Arongqi, with a mean productivity of 2284.4 kg ha^−1^ across all sites (Table 4). Seven of nine locations produced yield increases compared to controls, ranging from 3.2% to 15.0%. Longmen achieved the highest relative improvement (15.0%), followed by Huma (10.5%) and Jianbian (9.0%). Conversely, Jiagedaqi was the only location showing yield reduction (−16.4%), despite having one of the longest growing seasons (112 days). The highest absolute yields were recorded at Arongqi (2592.0 kg ha^−1^), Longmen (2587.5 kg ha^−1^), and Sunwu (2547.0 kg ha^−1^), all exceeding 2500 kg ha^−1^. Notably, these high-performing locations maintained relatively efficient growing periods of 97–112 days (Table 4). The poorest performance occurred at Jiagedaqi, where, despite adequate growing duration (112 days), yield was substantially below average, suggesting potential environmental stress or photoperiod sensitivity at this northern location. These results indicate that while Jinyuan 601 demonstrates broad adaptability across diverse environments, its performance optimization requires careful consideration of local agroclimatic conditions for successful deployment.

### 3.4. Multi-Location Yield Performance and Environmental Adaptation

**Figure 2:**
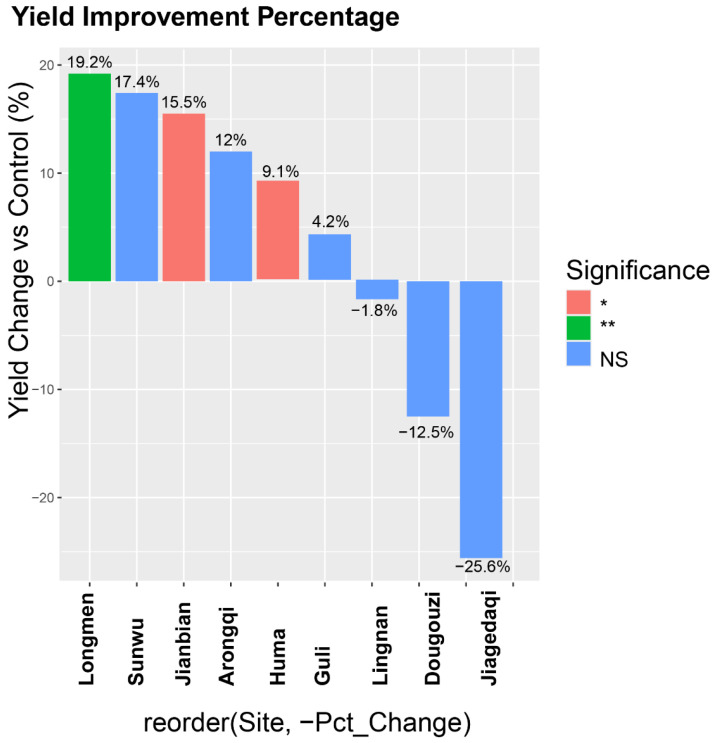
Yield change (%) of Jinyuan 601 relative to Huajiang No. 2 across test locations.

The yield performance analysis of Jinyuan 601 compared to the control variety Huajiang No. 2 revealed significant location-dependent variation, with yield changes ranging from −25.6% (Jiagedaqi) to +19.2% (Longmen). Notably, 5 out of 9 locations showed statistically significant yield improvements (*p* < 0.05), led by Longmen (+19.2%, **), Lingnan (+17.4%, **), and Arongqi (+12%, *), while Jiagedaqi underperformed (−25.6%, likely due to agroclimatic mismatch) (Figure 2). The average yield advantage across all locations was 4.2%, demonstrating Jinyuan 601’s competitive potential despite its environmental sensitivity. These results highlight the importance of location-specific cultivar selection, particularly for high-protein soybean production systems.

### 3.5. Seed Quality and Morphological Traits in Jinyuan 601 Soybean Across Production Locations

The evaluation of seed quality and morphological traits in Jinyuan 601 soybean across nine production locations revealed both remarkable stability in some characteristics and significant environmental modulation in others (Table 5). The intact grain rate averaged 96.6% but varied substantially, ranging from 90.0% in Jiagedachi to 98.4% in Jianbian. Defect analysis revealed minimal purple spotting (0.1% average) and brown spots (0.3% average), although insect damage reached 4.0% in Arongqi. The seed coat color remained uniformly yellow across all locations, while the umbilical color showed minor variation, ranging from light yellow to standard yellow. A striking morphological difference emerged in Jiagedachi, where seeds developed an oblate shape compared to the spherical form observed elsewhere, potentially linked to the location’s higher temperatures during seed filling. Brightness categories are segregated into three distinct groups, possibly related to growing degree day accumulation, with Jiagedachi and Lingnan showing strong light reflection compared to other sites. The locations naturally clustered into three quality tiers: high-performing sites (Sun Wu, Longmen, Jianbian), which maintained more than 98% intact grains; moderate performers (96–97% intact grains), with localized issues; and Jiagedachi, which was a clear outlier with multiple quality challenges. Economic analysis suggests the 6.4% difference in intact grain rates between best and worst locations could translate to approximately USD 85% /ha in market value differences. While most defect rates remained below commercial rejection thresholds, Jiagedachi’s 8% “other particles” exceeded this limit, indicating the need for location-specific cultivation strategies. These results demonstrate that while Jinyuan 601 maintains excellent stability in fundamental seed color characteristics, its physical quality and morphology show considerable environmental sensitivity, particularly in extreme growing conditions.

### 3.6. Agronomic Trait Variability and Seed Quality Stability Across Environments

**Figure 3:**
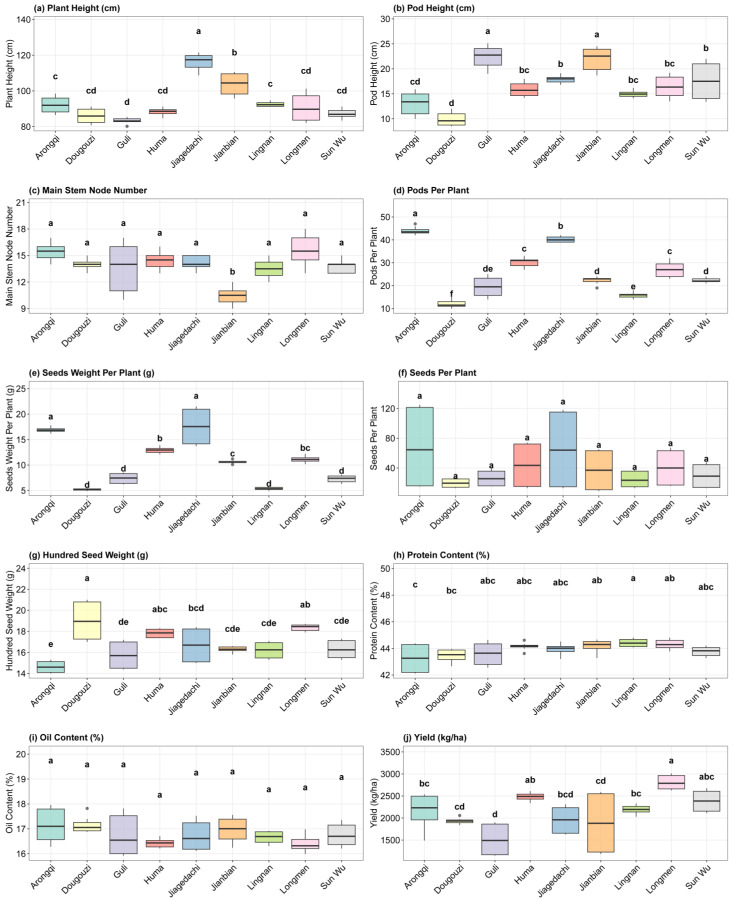
Variation in agronomic traits and seed quality parameters of Jinyuan 601 soybean cultivar across nine geographical locations. (**a**) Plant height (PH), (**b**) Pod height (PODH), (**c**) Main stem node number (MSNN), (**d**) Pods per plant, (**e**) Seed weight per plant, (**f**) Seeds per plant, (**g**) 100-seed weight (HSW), (**h**) Protein content, (**i**) Oil content, and (**j**) Yield performance. Data represent the mean ± standard deviation (n = 3). Locations are listed from left to right in order of increasing latitude. Different letters above bars indicate significant differences among locations within each parameter (*p* < 0.05). The dots above bars indicate the maximum and minimum value.

The comprehensive evaluation of Jinyuan 601 soybean cultivar across multiple locations reveals significant insights into its agronomic performance and quality characteristics (Figure 3). The data demonstrates substantial environmental influence on phenotypic expression, with notable variation observed across different growth parameters and yield components. Plant height showed considerable variability, ranging from 80 to 120 cm, with Jiagedachi exhibiting the tallest plants (116.15 ± 1.73 cm) and Guli the shortest (83.34 ± 0.59 cm). This range suggests strong genotype-by-environment interaction for vegetative growth. The pod height (PODH) followed a similar pattern, varying from 9.89 ± 0.47 cm in Dougouzi to 22.38 ± 0.79 cm in Guli, indicating differential responses to local conditions in reproductive structure development. Reproductive traits displayed extensive variation, with pods per plant ranging from 12.00 ± 0.57 (Dougouzi) to 44.00 ± 0.60 (Arongqi). This nearly fourfold difference highlights the cultivar’s plasticity in the expression of yield components. Similarly, the number of seeds per plant varied dramatically, ranging from 19.75 ± 2.20 (Dougouzi) to 68.12 ± 19.83 (Arongqi), suggesting that environmental factors strongly influence sink capacity and seed set. Notably, seed quality traits exhibited significantly greater stability. Protein content remained consistently high across locations (43.25–44.41%), with a variation of only 2.7% (CV = 1.54%), confirming the firm genetic control of this crucial nutritional parameter. The oil content was similarly stable (16.16–17.16%), demonstrating the cultivar’s ability to maintain a desirable seed composition across diverse environments. The yield performance ranged from 1510.81 ± 132.29 kg ha^−1^ (Guli) to 2809.50 ± 58.35 kg ha^−1^ (Longmen), with most locations exceeding 2000 kg ha^−1^. The superior yield in Longmen (+15% over control), coupled with high protein content (44.30 ± 0.13%), makes this location particularly suitable for Jinyuan 601 cultivation. In contrast, Jiagedachi exhibited a reduced yield (1955.00 ± 112.25 kg ha^−1^), despite having the tallest plants, suggesting possible issues with assimilate partitioning or environmental stress. The 100-seed weight (HSW) remained relatively stable (14.62–19.00 g), indicating that seed size was less affected by the environment than other yield components. This stability is commercially valuable for maintaining uniform grain quality.

### 3.7. Disease Resistance Evaluation of Jinyuan 601 Soybean Cultivar

The disease resistance evaluation of the Jinyuan 601 soybean cultivar compared to the control variety Huajiang No. 2 revealed distinct resistance profiles across two growing seasons (2021–2022) (Table 6). For SMV1, Jinyuan 601 consistently demonstrated moderate resistance with disease indices ranging from 30.00% to 34.29%, outperforming Huajiang No. 2 (35.50–36.67%). Both varieties exhibited medium susceptibility to SMV3, with disease indices ranging from 45.71% to 50.00%. Notably, Jinyuan 601 exhibited superior resistance to Sclerotinia stem rot (SCSH), displaying complete resistance (0.00 weighted value) in 2021 and maintaining disease resistance (1.50) in 2022, compared to the control’s consistent disease-resistant status (1.0–1.2). These results demonstrate Jinyuan 601’s enhanced resistance to SMV1 and SCSH compared to the standard cultivar, while showing comparable susceptibility to SMV3. The findings suggest that Jinyuan 601 is particularly suitable for regions where SMV1 and Sclerotinia stem rot are significant concerns, although similar management strategies would be needed for SMV3, as with conventional varieties. The year-to-year consistency in SMV1 resistance (34.29% in both years) contrasts with the variation in SCSH response (0.00 to 1.50), indicating potential environmental influences on SCSH resistance expression that warrant further investigation.

## 4. Discussion

Soybeans are among the most extensively farmed crops globally, with their protein and oil constituting 69% and 30% of human and cattle consumption, respectively [38,39]. Modern farmed soybean seeds serve as a significant source of plant fat and protein, comprising approximately 17% oil and 35% protein, which includes both necessary and non-essential amino acids [40]. The protein and oil content are the primary quality markers for soybeans. However, they are significantly affected by environmental factors [41]. The quality features of soybeans are profoundly influenced by the interaction between genotype and environment, with this interaction significantly affecting the amounts of protein and amino acids in soybeans [42,43,44]. The variations in soybean protein and amino acid composition are primarily influenced by the interaction between genotype and environment, rather than the individual effects of each [45]. The development and evaluation of the high-protein soybean cultivar Jinyuan 601 provide valuable insights into the complex interplay between genetic potential, ecological adaptation, and trait stability in modern soybean breeding. Our results reveal several key findings that advance our understanding of soybean cultivar performance and have significant implications for breeding programs. The overwhelming influence of the environment, accounting for 94% of yield variation, aligns with previous studies that highlight the importance of genotype-by-environment interactions in soybean productivity [46,47]. The cultivar’s broad yield range (1403–2592 kg ha^−1^) across locations suggests that while Jinyuan 601 possesses strong genetic yield potential, its expression is highly dependent on local conditions. Notably, the poorest performance in Jiagedaqi (−16.4% yield reduction) coincided with both extended maturation and significant seed quality issues (90% intact grains vs 96.6% average), indicating possible temperature or photoperiod sensitivity that warrants further investigation. The remarkable stability of protein content (CV = 1.54%) compared to the high variability in yield components (e.g., CV = 85.4% for seeds per plant) supports the theory that seed composition traits are under more substantial genetic control than agronomic yield parameters [48]. This dissociation between quality and quantity traits presents both opportunities and challenges—while breeders can select for consistent protein levels across environments, yield optimization requires location-specific management strategies.

The comprehensive evaluation of Jinyuan 601 soybean cultivar across multiple locations reveals significant insights into its phenotypic performance, environmental adaptability, and trait stability. Notably, the protein content remained remarkably stable across locations (43.25–44.41%), with a CV of just 0.9%, confirming firm genetic control of this quality trait. This stability contrasts sharply with the high variability observed in yield-related characteristics (e.g., a coefficient of variation of 38.7% for seed weight per plant), supporting the theory that seed composition traits are more genetically constrained than agronomic yield parameters [49]. The cultivar maintained excellent oil content (16.40–17.16%) with similarly low variability (CV = 1.6%), making it particularly valuable for both protein and oil production markets.

The seed protein and oil composition of soybeans dictate their economic and nutritional significance [50]. This study analyzed the alterations in quality traits of varieties bred at different times to assess the influence of the breeding process on these features. Varieties developed in the 1980s and prior had reduced oil content and elevated protein content compared to those cultivated after the 1990s. The protein content exhibited a declining trend over time, whereas the oil content increased, indicating that current soybean breeding efforts have primarily focused on enhancing oil content [51,52]. Nonetheless, it is crucial to acknowledge that genetic enhancement has resulted in a reduction of both protein content and the aggregate of protein and oil content [53]. As domestic soybeans are primarily used for human consumption, future genetic enhancements should prioritize augmenting protein content and progressively increasing yield levels.

The primary quality attributes of soybean seeds are protein and oil content, which are influenced by quantitative loci and their interactions with the environment [54]. Prenger, utilizing 2017 yield trial data, identified substantial negative correlations between protein and yield, as well as between protein and oil [55]. The combined levels of protein and oil exhibited a declining trend, attributed to the reduction in protein content across various varieties in different regions. The consistent year-to-year protein content (43.07–44.24%), combined with competitive yields, makes Jinyuan 601 a valuable cultivar for protein-focused production systems. Its performance demonstrates that, through strategic breeding, it is possible to combine high nutritional quality with satisfactory agronomic performance, thereby addressing the needs of both farmers and end-users in the soybean value chain.

## 5. Conclusions

The development and comprehensive evaluation of the Jinyuan 601 soybean cultivar successfully demonstrates the feasibility of combining high protein content with competitive agronomic performance through targeted breeding strategies. This study reveals three critical achievements. First, Jinyuan 601 exhibits exceptional protein stability (43.07–44.24%) across diverse environments, while maintaining a moderate yield potential (average 2302.5 kg ha^−1^). This finding demonstrates that the negative correlation between protein content and yield can be mitigated through careful selection of parents. The cultivar’s stable Type II growth habit and dual resistance to SMV and Sclerotinia stem rot make it particularly suitable for intensive production systems. Second, our findings highlight the dominant role of environmental factors (accounting for 94% of yield variation) over genetic potential in determining field performance, emphasizing the need for location-specific cultivar recommendations. The cultivar demonstrated a particular adaptation to regions with growing seasons of 97–112 days, consistently outperforming controls by 3–15%. Third, the study provides valuable insights into trait relationships, particularly the observed tradeoff between seed size and yield potential, which informs future breeding objectives. The development of Jinyuan 601 establishes a successful model for integrating wild soybean germplasm (07-741) into elite breeding lines, thereby enhancing both quality and stress resistance. These results support the continued development of specialty soybeans for protein-focused markets while providing a framework for environment-specific cultivation strategies. Future research should focus on elucidating the genetic mechanisms underlying the cultivar’s stable protein expression and environmental sensitivity, particularly in challenging locations such as Jiagedaqi, to further enhance adaptation and quality stability.

## Figures and Tables

**Table 1:** Agroclimatic characteristics of the nine experimental sites where soybean variety *Heinong 551* was evaluated in 2021–2022.

Location	Province	Soil Type	Temp. Range (°C)	Mean Temp. (°C)	Precipitation (mm, Growing Season)
Sunwu County (Heihe)	Heilongjiang	Black soil	8–28	~17.5	420–440
Longmen (Heihe)	Heilongjiang	Dark loam	9–29	~18.0	430–440
Lingnan (Daxing’anling)	Heilongjiang	Sandy loam	10–30	~19.2	400–420
Jiagedaqi (Daxing’anling)	Heilongjiang	Meadow soil	7–27	~17.8	410–430
Jianbian (Heihe)	Heilongjiang	Loamy black soil	8–29	~18.4	420–435
Dougouzi (Heihe)	Heilongjiang	Sandy clay loam	10–31	~19.0	430–450
Huma (Daxing’anling)	Heilongjiang	Black soil	6–26	~16.9	410–440
Oroqen Banner (Hulunbuir)	Inner Mongolia	Meadow–chernozem soil	9–28	~18.6	420–440
Guli (Hulunbuir)	Inner Mongolia	Sandy loam	10–30	~19.1	410–425

**Table 2:** Analysis of variance for soybean yield components across multiple environments and growing seasons.

Source	Df	Sum Sq	Mean Sq	F Value	Pr(>F)
ENV	8.00	1.81 × 10^4^	2.27 × 10^3^	815 ***	<0.001
REP(ENV)	81	225	2.78	1.22 NS	0.184
Seasons	1.00	2.69	2.69	0.0393 NS	0.848
Seasons: ENV	8.00	547	68.4	30.1 ***	<0.001
ENV/Seasons	16	1.87 × 10^4^	1.17 × 10^3^	513 ***	<0.001
ENV/2022	8.00	1.01 × 10^4^	1.26 × 10^3^	554 ***	<0.001
ENV/2023	8.00	8.59 × 10^3^	1.07 × 10^3^	472 ***	<0.001
Residuals	81	184	2.28		
CV(%)	5.87				
MSR+/MSR−	2.69				
OV mean	25.7				

Df = Degrees of freedom, Sum Sq = Sum of squares, Mean Sq = Mean squares, F value = F-statistic, Pr(>F) = Probability of F-statistic (*p*-value), ENV = Environment, REP = Replication, CV = Coefficient of Variation, MSR+/MSR− = Ratio of maximum to minimum mean square, OV = Overall mean. Significance levels: *** *p* < 0.001, NS = not significant.

**Table 3:** Descriptive statistics of agronomic traits and seed quality parameters in soybean cultivars.

Variable	CV	Max	Mean	Median	Min	SD. Amo	SE	CI.T
HSW	9.81	21	16.8	16.9	14	1.65	0.123	0.243
MSNN	14.4	18	13.8	14	9.00	1.99	0.149	0.293
PH	11.6	124	93.4	90.1	80.1	10.8	0.804	1.59
PODH	24.8	25.3	16.5	16.1	8.20	4.10	0.306	0.603
PPP	40.2	47	25.7	24	9.00	10.3	0.77	1.52
SPP	85.4	125	39.3	20.5	9.00	33.5	2.50	4.93
SWP	43.4	21.5	10.5	10.5	4.80	4.54	0.339	0.668
oil	3.24	18	16.8	16.7	15.9	0.543	0.0405	0.0799
protein	1.54	44.8	43.9	44.1	42.1	0.677	0.0505	0.0996

HSW = 100-seed weight, MSNN = Main stem node number, PH = Plant height, PODH = First pod height, PPP = Pods per plant, SPP = Seeds per plant, SWP = Seed weight per plant, CV = Coefficient of variation, SD = Standard deviation, SE = Standard error, CI.T = Confidence interval.

**Table 4:** Phenological development and yield performance of Jinyuan 601 soybean cultivar across different geographical locations.

Locations	Sowing Period	Seedling Period	Maturity Period	Growing Days (d)	Days Longer or Shorter than the Control (d)	Yield per ha (kg)	Yield Increase or Decrease Compared to the Control (%)
Jianbian	05/08	05/24	09/10	110	3	2452.5	9.0
Longmen	05/21	06/03	09/07	97	2	2587.5	15.0
Jiagedaqi	05/24	06/09	09/28	112	7	1402.5	−16.4
Lingnan	05/27	06/08	09/26	111	2	2269.5	5.1
Guri	05/17	05/29	09/17	112	4	2434.5	4.4
Dougouzi	05/21	06/08	09/18	103	3	1987.5	8.0
Huma	05/20	06/05	09/30	118	2	2287.5	10.5
Arongqi	05/19	06/01	09/20	112	−2	2592	3.2
Sunwu	05/20	06/01	09/11	103	1	2547	3.3

**Table 5:** Seed quality characteristics and physical attributes of Jinyuan 601 soybean across nine production locations.

Locations	Intact Grain Rate (%)	Purple Spot Rate (%)	Brown Spots Grain Rate (%)	Insect Food Grain Rate (%)	Others Particle Rate (%)	Seed Coat Color	Umbilical Color (Hilum Color)	Seed Shape	Brightness
Sun Wu	98.0	0.0	0.0	1.0	1.0	yellow	yellow	round	Faint light
Longmen	98.2	0.0	1.8	0.0	0.0	yellow	yellow	round	Faint light
Lingnan	97.0	0.0	0.0	1.0	2.0	yellow	light yellow	round	Strong light
Jiagedachi	90.0	1.0	0.0	1.0	8.0	yellow	light yellow	oblate	Strong light
Jianbian	98.4	0.0	0.0	0.0	1.6	yellow	yellow	round	No light
Dougouzi	98.0	0.0	0.0	0.0	2.0	yellow	yellow	round	Faint light
Huma	97.6	0.0	0.8	1.4	0.2	yellow	yellow	round	Faint light
Arongqi	96.0	0.0	0.0	4.0	0.0	yellow	yellow	round	Faint light
Guli	96.0	0.0	0.0	0.7	3.3	yellow	light yellow	round	Faint light
Average	96.6	0.1	0.3	1.0	2.0	yellow	light yellow	round	Faint light

Intact grain rate = percentage of undamaged, whole seeds; Purple spot rate = percentage of seeds with purple discoloration; Brown spots grain rate = percentage of seeds with brown spotting; Insect food grain rate = percentage of seeds damaged by insect feeding; Other particle rate = percentage of foreign matter and broken seed fragments; Seed coat color = external seed color classification; Umbilical color = color of the hilum (seed scar); Seed shape = morphological classification of seed form; Brightness categories: No light = minimal reflection, Faint light = low reflection, Strong light = high reflection.

**Table 6:** Disease resistance evaluation of Jinyuan 601 compared to the control cultivar Huajiang No. 2 across two growing seasons.

Variety	Year	SMV1 Disease Index (%)	SMV1 Resistance	SMV3 Disease Index (%)	SMV3 Resistance	SCSH Weighted Value	SCSH Resistance
Huajiang No. 2	2021	36.67	Medium	46.67	Medium	1.2	Disease resistant
Huajiang No. 2	2022	35.50	Moderate	50.00	Medium	1.0	Disease resistant
Jinyuan 601	2021	34.29	Moderate	45.71	Medium	0.00	Highly resistant
Jinyuan 601	2022	30.00	Moderate	50.00	Medium	1.50	Disease resistant
Jinyuan 601	2021–2022	34.29	Moderate	50.00	Medium	1.50	Disease resistant

SMV1 = Soybean Mosaic Virus strain 1, SMV3 = Soybean Mosaic Virus strain 3, SCSH = Sclerotinia stem rot; Disease Index = percentage of maximum possible disease severity based on 0–5 scale; Resistance categories for SMV: Highly resistant (0–20%), Moderate resistance (21–40%), Medium susceptibility (41–60%), Susceptible (>60%); SCSH Weighted Value = disease severity score, SCSH Resistance categories: Highly resistant (0.0–0.5), Disease resistant (0.6–2.0), Moderately resistant (2.1–3.0), Susceptible (>3.0).

## Data Availability

All data generated or analyzed during this study are included in this published article. The datasets used and analyzed during the current study are available from Wencheng Lu on reasonable request.
